# Brain imaging features of children with Hoyeraal‐Hreidarsson syndrome

**DOI:** 10.1002/brb3.2079

**Published:** 2021-03-18

**Authors:** Ming‐Jie Zhang, Ya‐Xian Cao, Hui‐Ying Wu, He‐Hong Li

**Affiliations:** ^1^ Department of Radiology Guangzhou Women and Children's Medical Center Guangzhou China

**Keywords:** cerebellar hypoplasia, DKC1, dyskeratosis congenita, Hoyeraal‐Hreidarsson syndrome, TINF2

## Abstract

**Objective:**

This study aimed to summarize the magnetic resonance imaging (MRI) and computed tomography (CT) features of the central nervous system (CNS) in children with Hoyeraal‐Hreidarsson syndrome.

**Methods:**

The imaging and clinical data of four children diagnosed with Hoyeraal‐Hreidarsson syndrome by clinical and laboratory tests in the Guangzhou Women and Children's Medical Center were gathered and analyzed retrospectively. The clinical manifestations and CNS imaging features of Hoyeraal‐Hreidarsson syndrome were summarized based on our results and a literature review.

**Results:**

Our results showed that delayed development, skin pigmentation, nail/toenail dystrophy, thrombocytopenia, and anemia are the most observed clinical presentations of Hoyeraal‐Hreidarsson syndrome. Important findings on CNS imaging showed that all patients had cerebellar hypoplasia, delayed myelination, hydrocephalus, brain atrophy, and calcification. The gene mutations in all cases were consistent with those of dyskeratosis congenita, including TINF2 mutations in three cases and DKC1 mutations in one case.

**Conclusion:**

Hoyeraal‐Hreidarsson syndrome is a severe variant of dyskeratosis congenita. Both DKC1 and TINF2 mutations can lead to the phenotypes of Hoyeraal‐Hreidarsson syndrome. In our study, CNS imaging revealed that cerebellar hypoplasia has an important diagnostic value for Hoyeraal‐Hreidarsson syndrome while delayed myelination, calcification of the parenchyma, brain atrophy, and hydrocephalus are also important findings on CNS imaging. Combining imaging features with clinical and laboratory indicators can assist the diagnosis of Hoyeraal‐Hreidarsson syndrome.

## Graphical abstract

This study aimed to summarize the magnetic resonance imaging (MRI) and computed tomography (CT) features of the central nervous system (CNS) in children with Hoyeraal‐Hreidarsson syndrome. Hoyeraal‐Hreidarsson syndrome is a severe variant of dyskeratosis congenita. Both DKC1 and TINF2 mutations can lead to the phenotypes of Hoyeraal‐Hreidarsson syndrome. In our study, CNS imaging revealed that cerebellar hypoplasia has an important diagnostic value for Hoyeraal‐Hreidarsson syndrome while delayed myelination, calcification of the parenchyma, brain atrophy, and hydrocephalus are also important findings on CNS imaging. Combining imaging features with clinical and laboratory indicators can assist the diagnosis of Hoyeraal‐Hreidarsson syndrome.

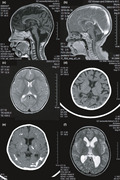

## 1 INTRODUCTION

Dyskeratosis congenita (DKC), also known as Zinsser‐Engman‐Cole syndrome, is a hereditary defect in telomere maintenance associated with telomerase dysfunction (Bessler et al., 2007). The incidence rate is approximately 1/10,000,000 cases, and the syndrome does not differ between males and females (Garcia et al., 2007). Gene mutations in patients with DKC lead to irregular telomere shortening in DNA (Alter et al., 2012; Gadalla et al., 2012), decreased telomerase activities, and impaired proliferation of hematopoietic stem cells, which gradually cause DKC (Calado & Young, 2008). Most DKC patients have short telomere lengths (Vulliamy et al., 2001) and are associated with 14 genes: ACD, DKC1, TERC, TERT, NOP10, NHP2, TINF2, USB1, TCAB1, CTC1, PARN, RTEL1, WRAP53, and C16orf57 (Islam et al., 2013; Kilic & Cekic, 2016; Pagon et al., 1993). DKC1, TERC, TERT, NOP10, NHP2, and TCAB1 encodes telomerase and TINF2 encodes the telomere protein complex. Three types of inheritance modes are presently recognized in this syndrome: X‐linked recessive inheritance caused by DKC1 mutations, autosomal dominant inheritance caused by TERT, TERC, and TINF2 mutations, and autosomal recessive inheritance caused by NOP10 mutations (Walne et al., 2007). These modes may have a variety of clinical manifestations and symptoms. For clinically suspected cases of DKC, the diagnosis is based on the discovery of pathogenic mutations in causal genes. The typical clinical characteristics of DKC include the triad of nail (toenail) dystrophy, skin pigmentation, and leukoplakia. Other clinical symptoms may include mental retardation, aplastic anemia (Vulliamy et al., 2005; Yamaguchi et al., 2005), malignant tumors, lung infection, idiopathic pulmonary fibrosis (Diaz de Leon et al., 2010), hepatic fibrosis (Yoshida et al., 2014), immunodeficiency, and scoliosis. The lowest standard of the diagnostic criteria for DKC is the presence of any two of the four major characteristics; the triad of nail (toenail) dystrophy, skin pigmentation, leukoplakia, and bone marrow failure and two or more other physical symptoms (Vulliamy et al., 2006).

Hoyeraal‐Hreidarsson syndrome is considered to be a severe variant form of DKC (Hoyeraal et al., 1970). Hoyeraal‐Hreidarsson syndrome can be diagnosed when a patient has cerebellar hypoplasia that is combined with some demonstrations of DKC (Sznajer, 2003) or any four of the six common characteristics, including intrauterine growth retardation, developmental delay, microcephaly, cerebellar hypoplasia, aplastic anemia, and immunodeficiency (Vulliamy et al., 2006). DKC1 mutations are normally considered to cause Hoyeraal‐Hreidarsson syndrome, but mutations in other genes, such as TINF2 mutations, may also manifest as Hoyeraal‐Hreidarsson syndrome (Dokal, 2011; Savage et al., 2008).

However, among the four cases of Hoyeraal‐Hreidarsson syndrome that has been recently diagnosed in our hospital, only one case was caused by DKC1 mutations while the other three cases were caused by TINF2 mutations. In this study, we performed a literature review and examined the four cases of Hoyeraal‐Hreidarsson syndrome to summarize the imaging features of the central nervous system (CNS) in children with Hoyeraal‐Hreidarsson syndrome.

## 2 MATERIAL AND METHODS

### 2.1 Demographic data and the patients' clinical examinations

The current study was approved by the Ethics Committee of our hospital, and all patients' legal guardians signed written informed consent.

Patient 1 was a 16‐month‐old male child. His parents sought treatment in our hospital 5 months after being diagnosed with developmental delay and thrombocytopenia.

Patient 2 was a five‐year‐old female child with a pale skin on her face. Her parents sought treatment in our hospital 2 years after being diagnosed with epistaxis and recurrent bleeding spots in her skin. At the time of her visit, she had been living with a developmental delay diagnosis for 3 years.

Patient 3 was a four‐year‐old male child. His parents sought treatment in our hospital 1 year after being diagnosed with developmental delay and thrombocytopenia.

Patient 4 was a five‐year‐old male child. When his parents sought treatment in our hospital, he had been living with a growth retardation diagnoses for 4 years, chronic diarrhea with mucus and blood in his stool for 3 years, and pancytopenia for 1 year.

A physical examination, hemograms, bone marrow biopsy, and genetic tests were completed for all patients.

### 2.2 Imaging examinations and methods

All four patients received cranial magnetic resonance imaging (MRI) examinations. Before the examinations were conducted, the patients' parents were fully informed about the side effects of the MRI examination and sedation before they signed informed consent. All pediatric patients were sedated with oral Chloral Hydrate at a dose of 0.5 ml/kg. MRI was started after the complete sedation was achieved. The scanner was a 1.5 T (Philips Achieva) MRI system. Transverse T1‐weighted imaging (T1WI), T2‐weighted imaging (T2WI), fluid‐attenuated inversion recovery (FLAIR) sequences, and sagittal T1WI sequences were acquired by conventional plain scans using fast spin‐echo (FSE) or turbo spin‐echo (TSE) sequences at a slice thickness of 6.0 mm, an interslice spacing of 1.0 mm, and a matrix size of 256 × 256. Enhanced transverse and sagittal T1WI sequences were acquired by contrast‐enhanced scans with the same parameter settings as the plain scans. The contrast agent for contrast‐enhanced scanning, gadolinium‐diethylenetriamine penta‐acetic acid (Gd‐DTPA), was intravenously injected at a dose of 0.2 ml/kg.

Patients 1 and 2 also received one plain cranial computed tomography (CT) scanning each. A Philips Brilliance 64 CT scanner was used for the CT scanning.

Two senior radiologists assessed the MRI and CT images independently and then collaboratively determined the final analysis.

## 3 RESULTS

### 3.1 The patients' clinical examination results

Patient 1: The physical examination showed no obvious irregularities in the nails and oral mucosa. A complete blood count showed thrombocytopenia and anemia. A bone marrow biopsy indicated the possibility of aplastic anemia. The genetic test results suggested missense mutations in the TINF2 gene (c.845G > A, p. Arg282His). Consequently, the patient was diagnosed with DKC.

Patient 2: The physical examination revealed dystrophy of the nails and toenails. A complete blood count showed thrombocytopenia and anemia. A bone marrow biopsy showed suspected aplastic anemia. The genetic test results suggested TINF2 mutations. Consequently, the patient was diagnosed with DKC.

**FIGURE 1:**
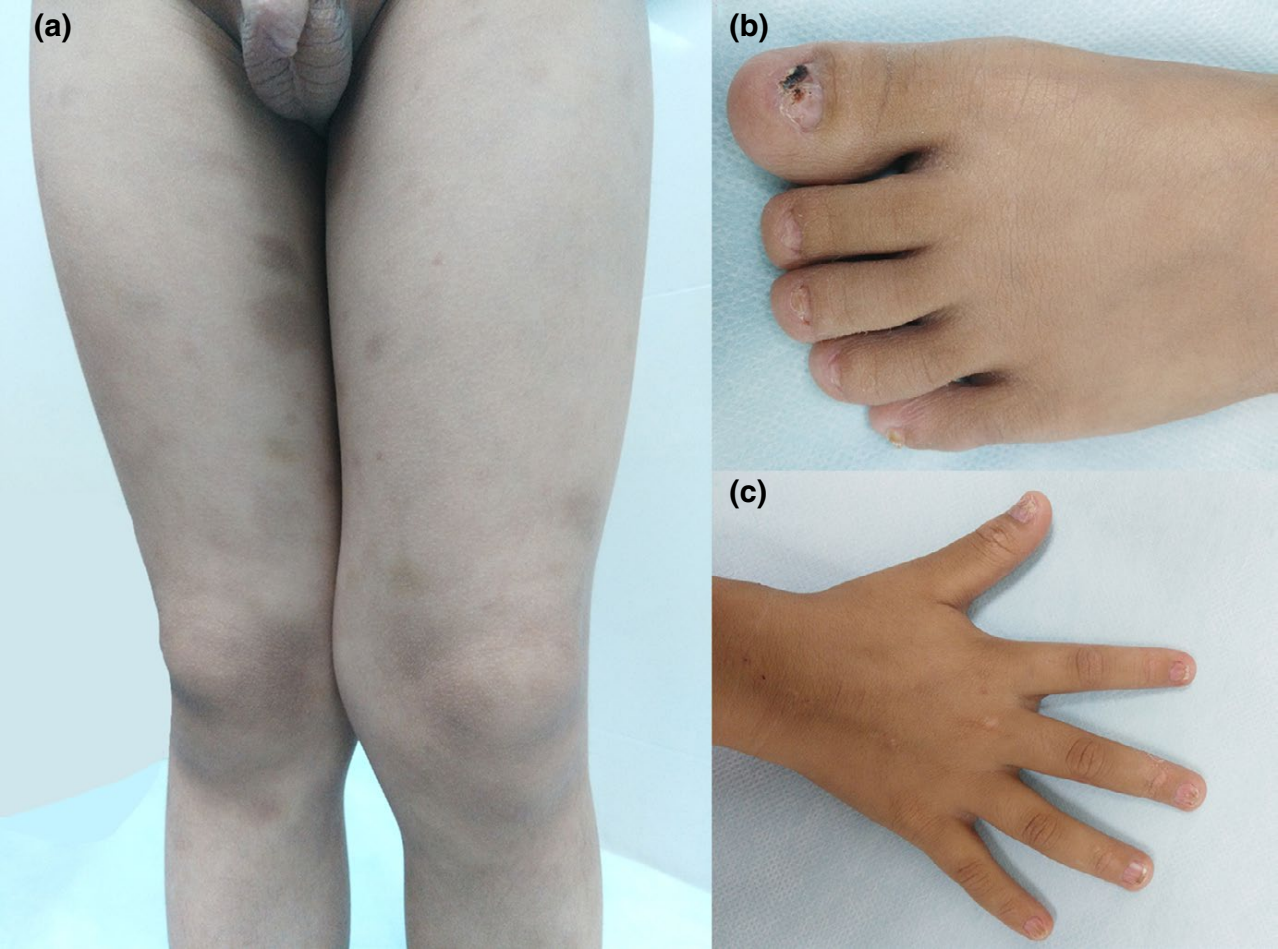
The physical examination of patient 3 revealed pigmentation macular is multiplex on the lower extremities and dystrophy of the nails and toenails. (a) Skin pigmentation and ecchymosis. (b) Toenail dystrophy. (c) Nail dystrophy

Patient 3: The physical examination revealed pigmentation macular is multiplex on the lower extremities and dystrophy of the nails and toenails (Figure 1). A complete blood count showed thrombocytopenia and anemia. A bone marrow biopsy showed hypoplasia of the bone marrow and suggested aplastic anemia. The genetic test results suggested missense mutations in the TINF2 gene (c.854G > A, p. Arg282His). Consequently, the patient was diagnosed with DKC.

Patient 4: The physical examination revealed that the pigmentation macular was multiplex on the lower back and lower extremities and mouth ulcers and lingual leukoplakia were observed. A complete blood count suggested pancytopenia. A bone marrow biopsy showed hypoplasia of the bone marrow and suggested aplastic anemia. The genetic test results suggested heterozygous mutations in the DKC1 gene (c.146C > T). Consequently, the patient was diagnosed with DKC.

### 3.2 The patients' imaging features

**FIGURE 2:**
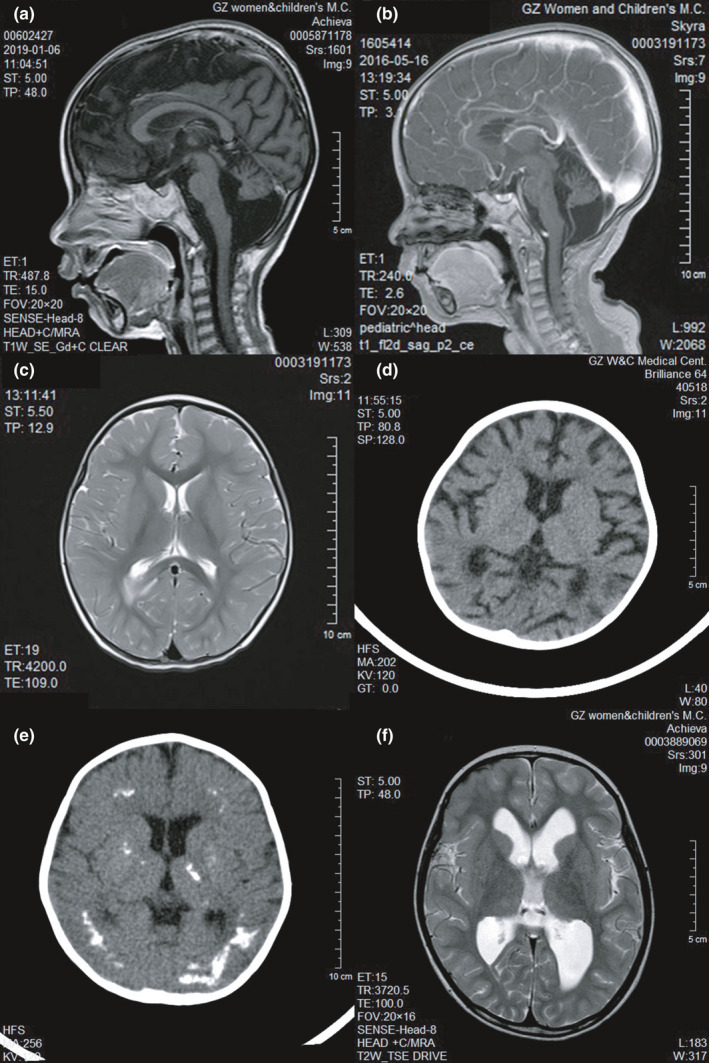
The patients' imaging features. (a) Patient 4, a median sagittal enhanced T1‐weighted image showing significant hypoplasia of the cerebellum with slightly enlarged fissure and enlargement of the posterior fossa. (b) Patient 3, cerebellar hypoplasia. (c) An axial T2‐weighted image of the bodies of the lateral ventricles in Patient 3 showing abnormal hyperintense signals in the white matter near the posterior horn of the right lateral ventricle and in the splenium of the corpus callosum. (d) An axial CT image of the horns of the lateral ventricles in Patient 4 showing deepening of the cerebral sulci in the two cerebral hemispheres and mild widening of the two lateral ventricles, suggesting brain atrophy. (e) A CT image of Patient 1 showing symmetrical, irregularly shaped calcifications in the two frontal lobes, the occipital lobe, and the basal ganglia. (f) An axial T2‐weighted image of Patient 2 showing dilation of the two lateral ventricles and the third ventricle, suggesting hydrocephalus

All four patients exhibited cerebellar hypoplasia involving the cerebellar hemispheres and cerebellar vermes. Sagittal T1WI showed a small cerebellum with a slightly enlarged fissure and that the distance between the lower edge of the cerebellum and the foramen magnum was significantly increased (Figure 2a,b).

Patient 3 had a myelination disorder. The myelination of his brain lagged behind that compared to normal children of the same age. MRI showed irregular signals in the white matter near the posterior horns of the lateral ventricles and in the splenium of the corpus callosum, which were hypointense on T1WI and hyperintense on T2WI (Figure 2c). The patients' corpus callosum was also thin.

Patient 4 exhibited brain atrophy. The sulci of the two cerebral hemispheres were deepened, the two lateral ventricles and the third ventricle were slightly widened, and no obvious irregular signals were observed in the brain parenchyma (Figure 2d).

Patient 1 showed calcification of the cerebral parenchyma. Cranial MRI showed extensive irregularities in the white matter signals of the two cerebral hemispheres, which manifested as multiple patchy hypointense signals mixed with extensive hyperintense signals on T2WI. Cranial CT confirmed that these hypointense signals on T2WI corresponded to the large‐scale calcified lesions (Figure 2e).

Patient 2 had hydrocephalus. The two lateral ventricles and the third ventricle were dilated (Figure 2f).

## 4 DISCUSSION

The central nervous system (CNS) findings are important manifestations of Hoyeraal‐Hreidarsson syndrome. MRI characteristics of the central nervous systems in patients with Hoyeraal‐Hreidarsson syndrome include cerebellar hypoplasia, delayed myelination, a thin corpus callosum, and a small pituitary gland, some of which may elicit hyperintense signals in the brain stem and the thalamus on T2WI, which indicate gliosis (Kuwashima, 2009). CT scans may reveal multiple scattered calcifications in the brain parenchyma.

Cerebellar hypoplasia is the most important characteristic of Hoyeraal‐Hreidarsson syndrome. In this study, the four pediatric patients had significant manifestations of cerebellar hypoplasia. MRI showed that the volumes of the cerebellar hemispheres and cerebellar vermes were smaller when compared to normal children of the same ages. The sagittal images showed that the space between the lower edge of the cerebellum and the foramen magnum was increased, which was partially filled by a large cerebrospinal fluid space. However, the morphologies of the cerebellar fissures were normal. Atrophy of the two cerebral hemispheres may also occur. MRI manifestations include the widening of the subarachnoid space, the deepening of sulci, the widening and deepening of the Sylvian cistern, and the mild widening of the two lateral ventricles and the third ventricle. Brain atrophy may be related to growth retardation, developmental delay, drug treatment effects, and secondary malnutrition. Therefore, cerebellar hypoplasia in patients with Hoyeraal‐Hreidarsson syndrome must be differentiated from cerebellar atrophy. The pathological basis of cerebellar hypoplasia is a decreased number of adequately sized cells while the pathological basis of cerebellar atrophy includes progressive cell loss. The imaging features of cerebellar atrophy include diffuse shrinkage of the cerebellar folia and an enlarged fissure (D'Arrigo et al., 2005).

Hoyeraal‐Hreidarsson syndrome must also be distinguished from Dandy–Walker syndrome (DWS), whose imaging features include hydrocephalus, the absence of the cerebellar vermis or the upward rotation of the residual vermis, and a very large posterior fossa cyst that links to the dilated fourth ventricle, though the morphologies of the two cerebellar hemispheres are normal. Patients with Hoyeraal‐Hreidarsson syndrome may display delayed myelination in the white matter. MRI revealed a few hyperintense signals on T2WI in the white matter near both lateral ventricles, an unclear boundary between the white and gray matter on T2WI, and inapparent subcortical white matter. Myelination in these patients lagged behind that compared to normal children of the same ages.

Calcification in the cerebral hemisphere is another significant manifestation of Hoyeraal‐Hreidarsson syndrome. In this study, one patient had multiple hyperintense signals in the thalamus, basal ganglia, brain stem, and brain white matter on T1WI and extensive hyperintense signals mixed with a small number of patchy hypointense signals in the white matter near the lateral ventricles on T2WI. CT scan showed a large number of calcifications in the thalamus, basal ganglia, and brain stem and in the white matter near the lateral ventricles. The underlying mechanisms in the development of calcifications in brain parenchyma are still unclear and may be related to dystrophy.

One patient in this study (Patient 2) had hydrocephalus that manifested as the dilation of the two lateral ventricles and the third ventricle, and this patient was the only female patient in this study. This finding, therefore, poses questions such as, if female dyskeratosis congenita (DKC) patients are prone to hydrocephalus. No DKC cases with similar symptoms have been reported in previous literature, and more cases are required to confirm this hypothesis.

Although the sample size in this study was relatively small, we consider that DKC instances in South China may be more likely to be related to TINF2 mutations and female DKC patients may be more prone to hydrocephalus. However, more cases are required to confirm these hypotheses.

## 5 CONCLUSION

Patients with a clinical diagnosis of dyskeratosis congenita (DKC), particularly those with TINF2 mutation associated DKC, should undergo routine CT and MRI examinations of the central nervous system (CNS). If the CT images show multiple calcifications in the brain parenchyma or the MRI images indicate cerebellar hypoplasia and delayed myelination, the possibility of a severe variant of dyskeratosis congenita (DKC), namely Hoyeraal‐Hreidarssonsyndrome, should be considered.

## CONFLICT OF INTEREST

None.

## AUTHOR CONTRIBUTION

Ming‐Jie Zhang and Ya‐Xian Cao were involved in drafting the manuscript and revising it critically for important intellectual content and conception and design; Hui‐Ying Wu made substantial contributions to acquisition and interpretation of data; He‐Hong Li analyzed the data; all authors given final approval of the version to be published.

### PEER REVIEW

The peer review history for this article is available at https://publons.com/publon/10.1002/brb3.2079.

## Data Availability

The datasets generated and/or analyzed during the current study are not publicly available due to the lack of an online platform but are available from the corresponding author on reasonable request.
